# Factors associated with SARS-CoV-2 infections among migrants in Germany: Carmen Koschollek

**DOI:** 10.1093/eurpub/ckac131.507

**Published:** 2022-10-25

**Authors:** C Koschollek, K Kajikhina, N Michalski, C Hövener

**Affiliations:** 1 Department Epidemiology and Health Monitoring, Robert Koch Institute, Berlin, Germany; 2 Department Infectious Disease Epidemiology, Robert Koch Institute, Berlin, Germany

## Abstract

**Background:**

International research shows increased risks for SARS-CoV-2 infection and severe disease progression in people with migration history. In Germany, data on this topic is scarce. Aim of this contribution is to examine the association between migrant status and risk of SARS-CoV-2 infection and discuss potential explanatory mechanisms.

**Methods:**

We analysed data from the German COVID-19 Snapshot Monitoring online-survey and performed hierarchical multiple regression models to calculate probabilities for a self-reported SARS-CoV-2 infection. Main predictor variable was the migrant status; besides, the association with gender, age, education, household size, household language (German vs. other), and occupation in the health sector was analysed.

**Results:**

Of 45,858 participants, 3.5% reported a current or previous infection with SARS-CoV-2, 16% reported own or parental history of migration. The probability of reporting an infection was 3.95 percentage points higher among migrants. The effect of different characteristics on self-reported SARS-CoV-2 infection varied. Higher probabilities were shown for individuals living in bigger households and those not speaking German at home. Stepwise integration decreased the observed association with migrant status. When adding an interaction term of migrant status and occupation in the health sector, the probability to report an infection was 11.5 percentage points higher for migrants working in the health sector.

**Conclusions:**

People with migration history, health sector employees and particular migrant health workers are at a higher risk of SARS-CoV-2 infection. However, the migrant status itself does not determine the risk of infection, but the living and working conditions. Therefore, targeted and multilingual prevention measures are needed that consider living and working conditions.

**Key messages:**

• Higher SARS-CoV-2 infection risks are not solely determined by migrant status, but were shown for health care workers, people living in bigger households and those not speaking German at home.

• As not the migrant status determines infection risks, multilingual and targeted prevention measures considering the living and working conditions of people are necessary.

